# Supplementary material for: Incidence and Risk Factors for Wound Revision after Surgical Treatment of Spinal Metastasis: A National Population-Based Study in South Korea
Source: Healthcare (Basel). 2023 Nov 15;11(22):2962. doi: 10.3390/healthcare11222962 (PMC10671392; doi:10.3390/healthcare11222962)
Supplement: Supplementary file 1 [file healthcare-11-02962-s001.zip › healthcare-2673753-supplementary.pdf]

Supplement.

Table S1. Korea Informative Classification of Diseases procedural codes of surgical treatment

Surgery	Korea Informative Classification of Diseases procedural codes
**Corpectomy**	N0450-N0453, 0464-0466, 2461-2466 S4704, 4706, 4708
**Instrumentation**	N0461, 0462, 0463, 0467-0470, 1460, 1466, 1469, 2467-2470
**Decompression**	N1491, 1492, 1493, 1497, 1498, 1499, N2497, 2498, 2499, S4705, 4707, 4709
**Vertebroplasty**	N0471, N0472, N0473, N0474

Table S2. ICD-10 codes for the Charlson comorbidity index

Disease	ICD-10 codes
**Myocardial infarction**	I21.x, I22.x, I25.2
**Congestive heart failure**	I09.9, I11.0, I13.0, I13.2, I25.5, I42.0, I42.5 - I42.9, I43.x, I50.x, P29.0
**Peripheral vascular disease**	I70.x, I71.x, I73.1, I73.8, I73.9, I77.1, I79.0, I79.2, K55.1, K55.8, K55.9, Z95.8, Z95.9
**Cerebrovascular disease**	G45.x, G46.x, H34.0, I60.x - I69.x
**Dementia**	F00.x - F03.x, F05.1, G30.x, G31.1
**Chronic pulmonary disease**	I27.8, I27.9, J40.x - J47.x, J60.x - J67.x, J68.4, J70.1, J70.3
**Rheumatic disease**	M05.x, M06.x, M31.5, M32.x - M34.x, M35.1, M35.3, M36.0
**Peptic ulcer disease**	K25.x - K28.x
**Mild liver disease**	B18.x, K70.0 - K70.3, K70.9, K71.3 - K71.5, K71.7, K73.x, K74.x, K76.0, K76.2 - K76.4, K76.8, K76.9, Z94.4
**Diabetes without chronic complication**	E10.0, E10.1, E10.6, E10.8, E10.9, E11.0, E11.1, E11.6, E11.8, E11.9, E12.0, E12.1, E12.6, E12.8, E12.9, E13.0, E13.1, E13.6, E13.8, E13.9, E14.0, E14.1, E14.6, E14.8, E14.9
**Diabetes with chronic complication**	E10.2 - E10.5, E10.7, E11.2 - E11.5, E11.7, E12.2 - E12.5, E12.7, E13.2 - E13.5, E13.7, E14.2 - E14.5, E14.7
**Hemiplegia or paraplegia**	G04.1, G11.4, G80.1, G80.2, G81.x, G82.x, G83.0 - G83.4, G83.9
**Renal disease**	I12.0, I13.1, N03.2 - N03.7, N05.2 - N05.7, N18.x, N19.x, N25.0, Z49.0 - Z49.2, Z94.0, Z99.2

**Moderate or severe liver disease**	I85.0, I85.9, I86.4, I98.2, K70.4, K71.1, K72.1, K72.9, K76.5, K76.6, K76.7
**AIDS/HIV**	B20.x - B22.x, B24.x
**Lung cancer**	C34
**Liver cancer**	C22
**Breast cancer**	C50
**Colorectal cancer**	C18–20
**Stomach cancer**	C16
**Prostate cancer**	C61
**Multiple myeloma**	C90
**Other**	C51–57, C60–68
